# Design considerations of an IL13Rα2 antibody–drug conjugate for diffuse intrinsic pontine glioma

**DOI:** 10.1186/s40478-021-01184-9

**Published:** 2021-05-17

**Authors:** Xiaolei Lian, Dina Kats, Samuel Rasmussen, Leah R. Martin, Anju Karki, Charles Keller, Noah E. Berlow

**Affiliations:** Children’s Cancer Therapy Development Institute, 12655 SW Beaverdam Road-West, Beaverton, OR 97005 USA

**Keywords:** Diffuse intrinsic pontine glioma, Antibody–drug conjugates, Immunotherapy, IL13Rα2, Pediatric cancer

## Abstract

Diffuse intrinsic pontine glioma (DIPG), a rare pediatric brain tumor, afflicts approximately 350 new patients each year in the United States. DIPG is noted for its lethality, as fewer than 1% of patients survive to five years. Multiple clinical trials involving chemotherapy, radiotherapy, and/or targeted therapy have all failed to improve clinical outcomes. Recently, high-throughput sequencing of a cohort of DIPG samples identified potential therapeutic targets, including interleukin 13 receptor subunit alpha 2 (IL13Rα2) which was expressed in multiple tumor samples and comparably absent in normal brain tissue, identifying IL13Rα2 as a potential therapeutic target in DIPG. In this work, we investigated the role of IL13Rα2 signaling in progression and invasion of DIPG and viability of IL13Rα2 as a therapeutic target through the use of immunoconjugate agents. We discovered that IL13Rα2 stimulation via canonical ligands demonstrates minimal impact on both the cellular proliferation and cellular invasion of DIPG cells, suggesting IL13Rα2 signaling is non-essential for DIPG progression in vitro. However, exposure to an anti-IL13Rα2 antibody–drug conjugate demonstrated potent pharmacological response in DIPG cell models both in vitro and ex ovo in a manner strongly associated with IL13Rα2 expression, supporting the potential use of targeting IL13Rα2 as a DIPG therapy. However, the tested ADC was effective in most but not all cell models, thus selection of the optimal payload will be essential for clinical translation of an anti-IL13Rα2 ADC for DIPG.

**Supplementary Information:**

The online version contains supplementary material available at 10.1186/s40478-021-01184-9.

## Introduction

Approximately 350 children per year in the United States are diagnosed with the high-grade glioma (HGG) subtype denoted diffuse intrinsic pontine glioma (DIPG), which represents 16% of all pediatric and young adult central nervous system tumors [17,22,52]. The prognosis for DIPG patients is dire, with median survival lingering under 1 year and fewer than 1% of DIPG patients surviving to 5 years[50]. DIPG presents in the pons region of the midbrain as tumor nodules interwoven amongst normal tissue, making surgical removal impossible [40]. After initiation, DIPG cells disseminate and can invade into multiple brain regions and even into the spinal cord [12,46]. Standard clinical care for DIPG consists of 54–60 Gy local field radiotherapy dosed over 6 weeks which temporarily improves patient neurological function and extends survival by 2–3 months [45], although resulting in lower quality of life due to negative long-term effects of radiation on pediatric brain function and development [8]. Despite investigation of multiple regimens based on chemotherapy, radiotherapy or next-generation targeted therapeutic agents, outcomes for DIPG remain essentially unchanged resulting in a disease marred by minimal long-term survivorship desperately in need of new targets and therapeutics [9,14,18,20,21,27,29,38]. Given the marginal advances in clinical outcomes for DIPG, recent efforts to improve translational research have focused on high-throughput sequencing of available cohorts of patient-derived DIPG tissue samples which resulted in discovery of new targets for therapeutic intervention, including interleukin 13 receptor subunit alpha 2 (IL13Rα2) [7].

IL13Rα2 is a cell-surface protein involved in regulation of interleukin 13 (IL-13) and interleukin 4 (IL-4) signaling [15]. IL13Rα2 directly binds IL-13 as a monomer to induce IL13RA2 signaling [23], and IL13Rα2 shows four orders-of-magnitude greater binding affinity for IL-13 than the alpha 1 receptor subunit (IL13Rα1) [37]. Conversely, IL13RA2 is capable of associating with and regulating response of IL-4 only by acting in concert with IL-4R [2]. IL13Rα2 signaling has been studied in the context of multiple diseases: IL13Rα2 activates the focal adhesion kinase (FAK, also called PTK2) and phosphatidylinositol-4,5-bisphosphate 3-kinase (PI3K) pathways through the family with sequence similarity 120A (FAM120A) scaffold protein [5] which mediates invasion and metastasis in colon cancer, IL-13 stimulation of IL13Rα2 activates Transforming Growth Factor Beta 1 (TGFβ1) and induces allograft fibrosis [19], and IL13Rα2 cooperates with epidermal growth factor receptor vIII (EGFRvIII) to promote the growth of glioblastoma multiforme (GBM) [43]. IL13Rα2 has previously been identified as a target of interest in numerous cancers, including malignant gliomas [28,30,51], pancreatic cancer [23], melanoma [44], ovarian cancer [32], and colon cancer [5]. IL13Rα2 has been explored clinically as both a CAR-T cell based therapy and as a phase III recombinant protein immunoconjugate target trial for adult glioma with promising outcomes [10,11,33,34], supporting further investigation of immunotherapies or immunoconjugate therapies targeting IL13Rα2 in other diseases with appropriate receptor expression patterns.

Antibody–drug conjugates (ADCs) are a novel class of immunoconjugate therapeutics which chemically link protein-specific antibodies with potent cytotoxic agents to target antigen-expressing cells with high specificity. Recently, FDA approved ADCs such as trastuzumab emtansine, trastuzumab emtansine [6], inotuzumab ozogamicin [31], and polatuzumab vedotin [49] are regarded as breakthrough therapeutics for breast cancer, Hodgkin’s lymphoma, and acute lymphoblastic leukemia respectively, which subsequently resulted in initiation of numerous ADC-based clinical trials. Foremost among design considerations for new ADCs is identifying the cell surface antigen target for the ADC, which requires both differentially elevated expression of the antigen target in tumor cells and internalization then degradation of the conjugated antibody to release the chemically-bound cytotoxic molecules [35].

In the context of DIPG, previous high-throughput transcriptome sequencing identified IL13Rα2 as a DIPG-selective genetic target versus patient-derived normal brain tissue samples (17.32-fold overexpression, p = 0.0003) [7] and immunohistochemical (IHC) staining demonstrated 12/17 samples (70.5%) were positive for IL13Rα2 of which 6/12 (50.0%) showed medium or strong expression [7]. Similar expression patterns of IL13Rα2 in DIPG and normal brain tissue have recently been independently confirmed [56]. Overexpression of IL13Rα2 cell surface protein in cancerous cells and relative absence in normal tissue meets the first criteria of ADC design consideration and demonstrated internalization and degradation of both IL-13 and anti-IL13Rα2 antibody through IL13Rα2 receptor binding [5,10,11,16,33,34] meets the second criteria, suggesting an anti-IL13Rα2 ADC may be a viable therapy for DIPG.

In this manuscript, we investigate the role of IL13RA2 in growth of DIPG and validate the potential of an anti-IL13Rα2 antibody–drug conjugate as a potential novel treatment for DIPG. The studies presented here demonstrate that direct stimulation of IL13Rα2 through canonical ligands induces minimal effect on either cellular proliferation or cellular invasion of DIPG cells. Conversely, cell viability assays demonstrate strong association between elevated IL13Rα2 expression and sensitivity to anti-IL13Rα2 ADC agents, affirming the potential of IL13Rα2 as a therapeutic target in DIPG.

## Materials and methods

### Cell models

The following five cell models and associated growth media were used in the experiments performed for this study:

1. DIPG-6 cultured in complete tumor stem medium (TSM), defined below.
2. DIPG-17 cultured in complete TSM.
3. DIPG-24 cultured in complete TSM.
4. SF-8628 cultured in Dulbecco’s Modified Eagle Medium (DMEM) (Cat #11995065; Thermo Fisher Scientific, Waltham, MA, USA) supplemented with 1% Penicillin/Streptomycin (P/S) (Cat #15140122, Thermo Fisher Scientific) and 10% fetal bovine serum (FBS) (Cat #26140079; Thermo Fisher Scientific)
5. CHLA-200 cultured in Iscove’s Modified Dulbecco’s Medium (IMDM) (Cat #12440061; Thermo Fisher Scientific) supplemented with 1% P/S, 10% FBS, 4 mM L-Glutamine (L-Glut) (Cat #25030081; Thermo Fisher Scientific), and 1× Insulin-Transferrin-Selenium (ITS) (Cat #41400045; Thermo Fisher Scientific)

Cell model SF-8628 was also genetically modified to stably express luciferase and red fluorescent protein (RFP) using EF1a-Luciferase (firefly)-2A-RFP lentiviral particles (Cat # LVP440; GenTarget Inc, San Diego, CA, USA), which were subsequently puromycin selected to isolate a purified luciferase/RFP positive cell population. All cell cultures were maintained in Nunc EasyFlask T75 (Cat #156,499; Thermo Fisher Scientific) vented flasks stored in incubators set at 5% CO2 at 37 °C. Complete TSM is based on a previously published medium recipe for generation of patient-derived DIPG cell cultures [36]. Basal TSM consists of 250 mL Neurobasal-A (NBA) (Cat #10888022; Thermo Fisher Scientific), 250 mL Dulbecco's Modified Eagle Medium/Nutrient Mixture F12 (DMEM/F12) (Cat #11330032; Thermo Fisher Scientific), 1× antibiotic–antimycotic (Cat #15240096; Thermo Fisher Scientific), 2 mM GlutaMAX (Cat #35050061; Thermo Fisher Scientific), 10 mM HEPES (Cat #15630080; Thermo Fisher Scientific), 1 mM sodium pyruvate (Cat #11360070; Thermo Fisher Scientific), and 1× MEM non-essential amino acids (Cat #11140050; Thermo Fisher Scientific).

To create complete TSM media, basal TSM media is supplemented with 1× B-27 (Cat #12587010; Thermo Fisher Scientific), 2 µg/mL Heparin (Cat #07980; StemCell Technologies, Vancouver, BC, CA), 20 ng/mL recombinant human epidermal growth factor (EGF) (Cat #100-26; Shenandoah Biotechnology, Warwick, PA, USA), 20 ng/mL recombinant human basic fibroblast growth factor (FGF-basic) (Cat #100-26; Shenandoah Biotechnology), 10 ng/mL recombinant human platelet-derived growth factor glycoprotein A (PDGF-AA) (Cat #100-16; Shenandoah Biotechnology), and 10 ng/mL recombinant human platelet-derived growth factor glycoprotein B (PDGF-BB) (Cat #100-18; Shenandoah Biotechnology).

Additional details for cell models used in this study, including IL13Rα2 expression status, are provided in Table 1. The human alveolar rhabdomyosarcoma cell line RH30 (accession ID CVCL_0041) was used solely for a supplemental experiment to validate the recombinant IL-4 and IL-13 proteins used in this study. RH30 was cultured in Roswell Park Memorial Institute (RPMI) 1640 Medium (Cat #12633012; Thermo Fisher Scientific) supplemented with 1% P/S and 10% FBS. All cell lines were tested for authenticity at their institute of origin and were additionally authenticated at initiation of study via short tandem repeat (STR) fingerprinting (Additional file 3: Table 1) performed by the University of Arizona Genetics Core, as well as being surveyed for changes to proliferation, morphology, or behavior.

**Table 1:** DIPG and GBM cell models used in studies

Article Model Name	DIPG-6	DIPG-17	DIPG-24	SF-8628	CHLA-200
Official Name	SU-DIPG-VI	SU-DIPG-XVII	SU-DIPG-24	SF8628	CHLA-200
Accession ID	CVCL_IT40	N/A	N/A	CVCL_IT46	CVCL_M147
IL13Rα2 Status	IL13Rα2-High	IL13Rα2-High	IL13Rα2-Low	IL13Rα2-High	IL13Rα2-High
Model Type	Neurosphere Culture (suspension)	Neurosphere Culture (suspension)	Neurosphere Culture (generally adherent)	Cell Line (adherent)	Cell Culture (adherent)
Growth Media	Complete TSM	Complete TSM	Complete TSM	DMEM + 1% P/S + 10% FBS	IMDM + 1% P/S + 20% FBS + 4 mM L-Glut + 1× ITS
Age at diagnosis (years)	7	8	6	5	12
Gender	Female	Male	Female	Female	Male
Histology	HGG	DIPG	DIPG	DIPG	GBM
H3 status	H3.3K27M	H3.3K27M	H3.3K27M	H3.3K27M	Unknown
Grade	III	IV	IV	IV	IV
Origin	Pons	Pons	Pons	Unknown	Parietal lobe
Treatment	XRT + vorinostat	XRT + bevacizumab; panobinostat—> XRT—> everolimus	XRT + bevacizumab	Unknown	XRT + chemotherapy
Survival (months)	6	13	8	Unknown	Unknown
Tissue Source	autopsy	autopsy	autopsy	surgical biopsy	autopsy
Material Source	Monje Lab	Monje Lab	Monje Lab	EMD Millipore	COG Repository
PMID	25939062[25]	28823557[47]	28823557[47]	23603901[13]	22120608[54]

### Immunoblotting

Cells for immunoblotting were lysed in RIPA lysis and extraction buffer (Cat #89901; Thermo Fisher Scientific) supplemented with HALT protease and phosphatase inhibitor cocktail (Cat #78440; Thermo Fisher Scientific) following the manufacturer’s protocol. Collected protein lysates were stored at – 80 °C. 20 µg of protein was loaded into the channel of an SDS–polyacrylamide gel electrophoresis (PAGE) gel, one channel per cell model. Electrophoresis-separated proteins were electrically transferred to PVDF membrane, washed in TBST buffer, and blocked in 5% fat-free milk for 2 h at room temperature, then incubated with primary antibodies at 4 °C overnight. Following primary antibody blocking, specific signal was detected with species-appropriate horseradish peroxidase-conjugated secondary antibody using Clarity Western blot ECL Substrate (Cat #1705061; Bio-Rad Laboratories, Hercules, CA, USA) and imaged using an IVIS Lumina II imaging system (Caliper Life Sciences, Hopkinton, MA, USA).

The following primary-secondary antibodies were used in the study: anti-IL13Rα2 2E10 clone primary (Cat #WH0003598M1; Sigma-Aldrich, St. Louis, MO, USA) with Horse anti-Goat secondary (Cat #PI-9500; Vector Laboratories, Burlingame, CA, USA), rabbit anti-STAT6 (D3H4) primary antibody (Cat# 5397S; Cell Signaling Technology, Danvers, MA, USA) with goat anti-rabbit IgG secondary (Cat #PI-1000; Vector Laboratories), rabbit anti-pSTAT6 primary antibody (Cat #9361S, Cell Signaling Technology) with goat anti-rabbit IgG secondary, and anti-GAPDH primary (Cat #PLA0302; Sigma-Aldrich) with horse anti-mouse secondary (Cat #PI-2000; Vector Laboratories).

Western blot studies were completed in triplicate to ensure validity of protein expression trends.

### Ligand stimulation cell proliferation studies

Cells in single-cell suspension were plated in a 96-well plate (Cat #136101; Thermo Fisher Scientific) at 50 µL/well model-specific starvation medium. SF-8628 was plated in DMEM + 1% P/S at 500 cells/well, DIPG-24 was plated in TSM + 1× B27 + 2 µg/mL Heparin at 1500 cells/well. Cells were starved overnight, then supplemented with recombinant human IL-4 (Cat #100-09; Shenandoah Biotechnology) or recombinant human IL-13 (Cat #100-85; Shenandoah Biotechnology) to final concentrations of 0.5 ng/mL, 1 ng/mL, 5 ng/mL, 10 ng/mL, 50 ng/mL, and 100 ng/mL at a final volume of 100 µL/well. Negative control wells were supplemented 50 µL/well starvation medium (−CTRL), positive control wells were supplemented with 50 µL/well double concentration growth media to reach final concentration of 1× growth media (+ CTRL). Cells were incubated for 72 h then assayed with CellTiter Glo Luminescent Cell Viability Assay (Cat #G7570, Promega, Madison, WI, USA) per the manufacturer’s protocol. Luminescence was measured using a BioTek Synergy HT plate reader (BioTek, Winooski, VT). Cell growth was plotted in GraphPad Prism.

All stimulation experiments were performed with in triplicate for each treatment condition, with each experiment performed with three technical replicates for each cell model.

### IL-4 and IL-13 recombinant protein validation studies

RH30 cells were plated in 6 cm plates and serum starved overnight in RPMI-1640 media with 1% P/S. Cells were then exposed for 60 min to 20 ng/mL recombinant IL-4 in RPMI-1640 + 1% P/S, 20 ng/mL recombinant IL-13 in RPMI-1640 + 1% P/S, or RPMI-1640 + 1% P/S (control). Cells were then harvested as described in the Immunoblotting section and probed for overall STAT6 level and phosphor-STAT6 level to confirm phosphorylation of STAT6 following IL-4 or IL-13 exposure, similar to a previously published study [26]. Validation stimulation experiments were performed in triplicate for each treatment condition.

### Cell invasion studies

6.5 mm transwell inserts with 8 µm pores (Cat #662638; Greiner Bio-One, Kremsmünster, Austria) were treated 0.1 mL of 300 µg/mL Matrigel (Cat #35423; Corning Life Sciences, Tewksbury, MA, USA), placed in 24-well cell culture plates (Cat #662160; Greiner Bio-One), and incubated for 30 min at 37 °C. Next, cell models suspended in 300 µL starvation media at specific cell populations were plated into each insert: SF-8628 at 50,000 cells/insert, CHLA-200 at 50,000 cells/insert, and DIPG-24 at 100,000 cells/insert. The lower wells were plated with 600 µL media with the following experimental conditions: starvation media (-CTRL): SF-8628, DMEM + 1% P/S; DIPG-24, TSM + 1× B-27 + 2 µg/mL Heparin; IMDM + 1× ITS + 2× L-Glutamate + 1% P/S (-CTRL), recombinant human IL-13 at concentrations of 20 ng/mL, 50 ng/mL, 100 ng/mL final concentration, or growth media (+ CTRL). Plates were then incubated for 72 h at 37 °C with 5% CO_2_.

The inserts were removed, aspirated, washed with twice with H_2_O and PBS, and the inside of the inserts were swabbed to remove non-invasive cells. Remaining cells were fixed with 4% paraformaldehyde (PFA) for 20 min then washed with PBS and DDH_2_O. Fixed cells were stained with 0.1% crystal violet in 10% methanol for 20 min, washed twice with DDH_2_O, then allowed to dry overnight. Fixed, dried inserts were treated with 250 µL of 10% acetic acid and then crystal violet intensity was quantified using a BioTek Synergy HT plate reader at 595 nm read wavelength. Cell invasion was plotted in GraphPad Prism.

All invasion experiments were performed in triplicate for each treatment condition, with each experiment performed with three technical replicates for each cell model.

### Primary antibody-secondary antibody–drug conjugate cell viability studies

Cells suspended in appropriate growth media were plated into 96-well plates at 100 µL/well at specific populations: SF-8628 at 750 cells/well, CHLA-200 at 750 cells/well, DIPG-6 at 1500 cells/well, DIPG-17 at 1500 cells/well, and DIPG-24 at 1500 cells/well. Plates were allowed to rest overnight, then exposed to 15 ng/mL anti-IL13Rα2 2E10 primary antibody, incubated at 37°c with 5% CO_2_ for 10 min, and finally treated with Fragment antigen-binding (Fab) fragments of anti-mouse IgG fragment crystallizable (Fc) specific antibody conjugated to duocarmycin DM via non-cleavable linker (Fab-αMFc-CL-DMD) (Cat #AM-202-DD; Moradec LLC., San Diego, CA, USA) in the following concentrations: 0.2 pg/mL, 0.9 pg/mL, 4.8 pg/mL, 24 pg/mL, 120 pg/mL, 600 pg/mL, 3 ng/mL, and 15 ng/mL. Plates were incubated at 37°c with 5% CO_2_ for 120 h then assayed with CellTiter Glo Luminescent Cell Viability Assay per the manufacturer’s protocol. Luminescence was measured using a BioTek Synergy HT plate reader. Cell viability versus ADC concentration was plotted and IC_50_ values were calculated using GraphPad Prism.

All cell viability experiments were performed in triplicate for each treatment condition, with each experiment performed with three technical replicates for each cell model.

### Antibody Conjugations

Antibody conjugation was performed by Moradec, LLC. IL13Rα2 2E10 antibody was concentrated to approximately 2 mg/mL. 0.1 mL of concentrated antibody was used for initial conjugation optimizations to reach drug-antibody ratio (DAR) around 3–4, then the remaining antibody was used to carry out the conjugation with maleimide-valine-alanine-Pyrrolobenzodiazepine (Mal-VA-PBD). The conjugation was a maleimide-cysteine based method by first reducing the antibody inter-chain disulfide bonds with TCEP and then linking the maleimide moiety of the drug to the reduced cysteines to generate the ADC. The conjugated ADC was desalted on Sephadex G50 columns to remove residual unreactive toxins and then buffer exchanged pH 7.2 in PBS. The final DAR was calculated from the A333nm:A280nm ratio and was determined to be 3.36 for anti-IL13Rα2::PBD.

### Antibody–drug conjugate in vitro cell viability studies

Cells suspended in appropriate growth media were plated into 96-well plates at 150 µL/well at specific populations: SF-8628 at 750 cells/well, CHLA-200 at 750 cells/well, DIPG-6 at 1500 cells/well, DIPG-17 at 1500 cells/well, and DIPG-24 at 1500 cells/well. Plates were incubated overnight, then exposed to anti-IL13Rα2::PBD ADC at the following concentrations: 0 ng/mL, 1 ng/mL, 10 ng/mL, 20 ng/mL, 100 ng/mL, 200 ng/mL, 1 µg/mL, 2 µg/mL, 5 µg/mL, 10 µg/mL. Plates were incubated at 37°c with 5% CO_2_ for 144 h then assayed with CellTiter Glo Luminescent Cell Viability Assay per the manufacturer’s protocol. Luminescence was measured using a BioTek Synergy HT plate reader. Cell viability versus ADC concentration was plotted and IC_50_ values were calculated using GraphPad Prism.

All cell viability experiments were performed in triplicate for each treatment condition, with each experiment performed with three technical replicates for each cell model.

### Antibody–drug conjugate ex ovo cell viability studies

Fertilized *Coturnix japonica* (Japanese quail) eggs were purchased from Boyd’s Bird Company (Eagle Creek, OR, USA) and Purely Poultry (Fremont, WI, USA), stored at 4 °C for 120 h, and then incubated at 37 °C and 70% humidity for approximately 54 h in an egg incubator. Quail eggs were opened by our mechanical device [48] into six-well plates (Cat # CC Plate–PS-6A-F-C-S; Advangene, Lake Bluff, IL, USA), then incubated for 112 h (embryonic day 7) at which point unfertilized/non-viable quail were removed.

Cultured and trypsinized luciferase/RFP-expressing SF-8628 cells were added to hydrogel-c (Cat #GS313; ESI-BIO, Alameda, CA, USA) to a cell concentration of 5,000 cells/µL. Anti-IL13Rα2::PBD ADC carried in PBS was added to SF-8628-containing hydrogel, to final ADC concentrations of 0 ng/mL (PBS control), 50 ng/mL (n = 7 quail), 500 ng/mL (n = 7 quail), 5 µg/mL (n = 10 quail), and 50 µg/mL (n = 8 quail). 25 µL of each treatment concentration was added to 6.5 mm diameter sterilized fiberglass 3D meshes (Cat #SC-S510-0001; LenaBioscience, Atlanta, GA, USA) according to manufacturer’s protocol then incubated for approximately 30 min at 37 °C and 100% humidity.

The chorioallantoic membrane (CAM) of viable quails were superficial injured by carefully placing and carefully removing an Eppendorf tube lid against the CAM. The mesh tumor modules were placed on the injury site and incubated for 120 h. Add the end of the incubation, 100 µl of PBS containing 1.5 mg of luciferin-d (Cat #122799; PerkinElmer, Waltham, MA, USA) was added to the tumor modules, incubated in the dark for 5 min, and resulting bioluminescence was measured using an IVIS Lumina (PerkinElmer). The quail were imaged with a 15 s exposure for total light emission. Total light emission amongst ADC treatment concentrations were normalized to control emission to determine final SF-8268 cell viability.

### Statistics

Dunnett's multiple comparisons test performed in GraphPad Prism was used to analyze statistical differences in cytokine stimulation cell proliferation assays and in cytokine stimulation cell invasion assays. Testing for linear trend was performed in GraphPad Prism to determine if cell proliferation by cytokine stimulation occurred in a dose-dependent manner.

## Results

### IL13Rα2 is expressed in DIPG tumor samples and cell models

**Fig. 1:**
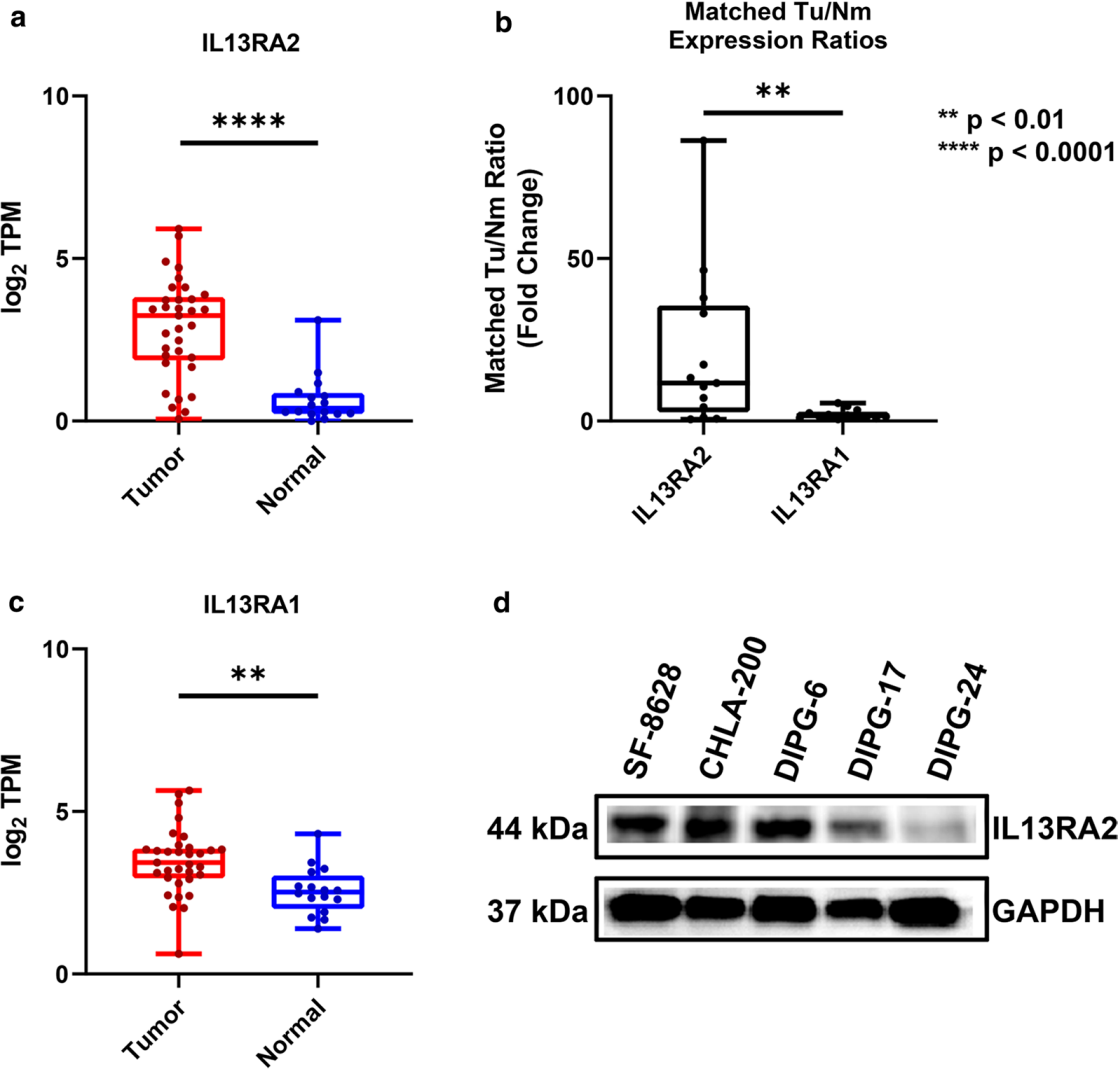
Gene expression in DIPG. High-throughput RNA sequencing of 33 DIPG tumor samples vs. 20 normal brain samples across two cohorts. **a** IL13Rα2 tumor expression versus normal expression (p < 0.01). **b** Tumor-normal expression ratios of IL13Rα2 and IL13Rα1 across patient-matched DIPG samples (p < 0.01). **c** IL13Rα2 tumor expression versus normal expression (p < 0.0001). **d** Expression of IL13Rα2 in DIPG and GBM cell models (HEK293 as positive control) determined by immunoblotting. SF-8628, CHLA-200, DIPG-6, and DIPG-17 are IL13Rα2-high models, while DIPG-24 is IL13Rα2-low

We expanded the cohort of sequenced DIPG transcriptomes by incorporating newly-published RNA sequencing data from ten (10) DIPG tumor samples and two (2) normal pons tissue samples [41]. Evaluation of the expanded cohort confirmed the status of IL13Rα2 overexpression, with the combined new dataset and previous dataset (n = 28 DIPG tumor samples, n = 18 normal tissue samples) demonstrating 21-fold overexpression in matched tumor vs. normal brain tissue and 12-fold overexpression in all tumor vs. normal samples (p < 0.0001) (Fig. 1a, b), remaining significantly higher (p = 0.01) than the tumor-normal expression ratio of IL13Rα1 (Fig. 1b, c).

Subsequently, available DIPG cell models were assayed for protein-level expression of IL13Rα2 to determine optimal models for further in vitro investigation. A list of cell models used in this study are provided in Table 1. Several cell models show strong IL13Rα2 expression, including CHLA-200, DIPG-6, DIPG-13, DIPG-17, and SF-8628 (Fig. 1d), while DIPG-24 shows notably lower (but not absent) IL13Rα2 expression.

### Functional impact of IL13Rα2 in DIPG growth and invasion

**Fig. 2:**
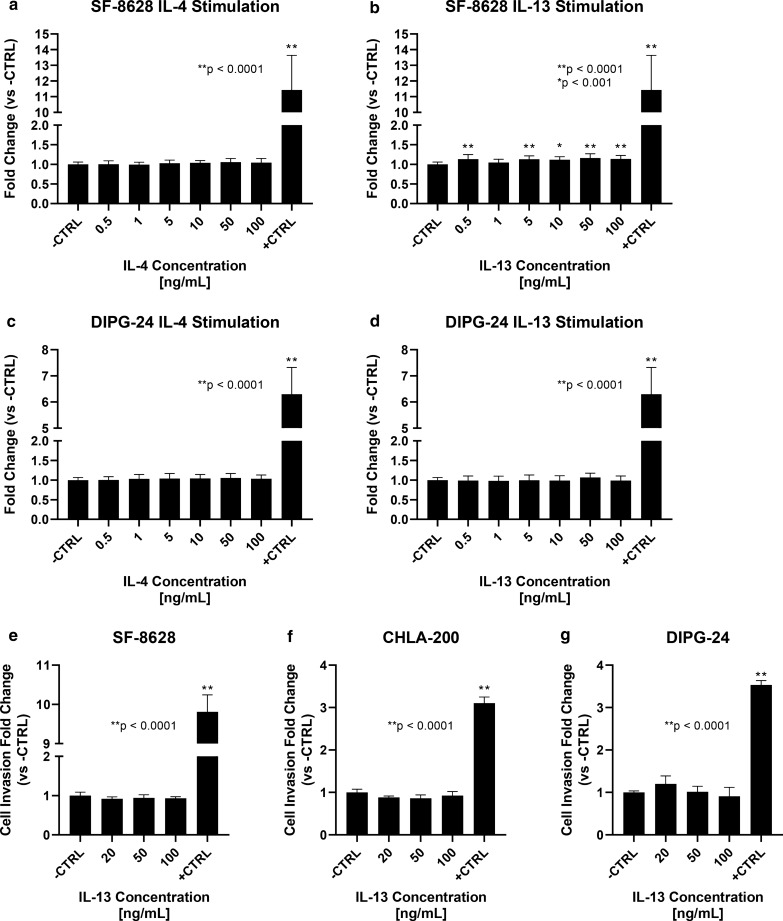
IL13Rα2 ligand stimulation growth and invasion assay results. Cell models were stimulated with IL13Rα2 ligands to determine effect of IL13Rα2-mediated signaling on cell proliferation and invasion. In a-d, cells were stimulated with recombinant IL-4 and IL-13 at concentrations between 0.5 and 100 ng/mL. In e–g, cells were stimulated with recombinant IL-13 at concentrations at 20 ng/mL, 50 ng/mL, and 100 ng/mL. **a** SF-8628 stimulated with recombinant human IL-4. **b** SF-8628 with recombinant human IL-13. **c** DIPG-24 stimulated with recombinant human IL-4. **d** DIPG-24 stimulated with recombinant human IL-13. **e** SF-8628 cellular invasion following IL-13 stimulation. **f** DIPG-24 cellular invasion following IL-13 stimulation. **g** CHLA-200 cellular invasion following IL-13 stimulation

Given the overexpression of IL13Rα2 and the importance of the receptor in other diseases, we first investigated the role of IL13RA2 signaling in DIPG. To determine if cytokine stimulation modifies cell growth in vitro, cells were serum- or supplement-starved for 24 h then treated with varying concentrations of the known cytokine binding partners of IL13Rα2, IL-4 and IL-13 (Fig. 2a–d). Of the cell model-cytokine combinations tested, only SF-8628 stimulated with IL-13 demonstrated any significant increase in cell growth versus starvation media as the negative control (Fig. 2b). Notably, the increased cell growth from IL-13 stimulation of SF-8628 was statistically dose-dependent (slope = 0.0215, p < 0.0001). All other combinations demonstrated no statistically significant change in cell viability (Fig. 2a, c, d). Lack of DIPG-24 response is consistent with the lower expression of IL13Rα2 in the assayed cell models, as DIPG-24 has reduced expression compared to SF-8628. However, no cell model showed increase in growth comparable to standard growth media (+ CTRL). While IL-13 may impact growth rate of a single cell model the effect is negligible compared to optimal growth conditions, and IL-4 demonstrated no significant impact on growth. We confirmed expected mechanistic activity of the IL-4 and IL-13 recombinant proteins by replicating a previously published study [26] (Additional file 2: Fig. 2).

Development of new DIPG tumor nodules both within the pons and throughout the CNS is critical to disease progression and mortality, thus we also investigated the effect of IL-13 stimulation on the invasive potential of DIPG/GBM cell models. As IL13Rα2 is significantly more selective for IL-13 and because IL-13 demonstrated a modest but statistically significant impact on growth versus starvation media, we focused on the impact of IL-13 on cellular invasion[37]. We assayed two IL13Rα2-high cell models (SF-8628 and CHLA-200) and one IL13Rα2-low cell model (DIPG-24) to determine if IL-13 stimulation impacted cellular invasion in a dose-dependent manner (Fig. 2e–g). Neither the IL13Rα2-high nor IL13Rα2-low cell models showed any statistically significant change in cell invasion following IL-13 stimulation versus negative control. Conversely, cells treated with standard growth media (+ CTRL) demonstrated a statistically significant increase in invasion, showing the propensity for the cell models to invade in proper conditions.

### Pharmacological testing of IL13Rα2-targeting therapeutics in DIPG

Having determined that exposure to canonical IL13Rα2 ligands resulted in minimal impact on cell proliferation and invasion, we investigated the pharmacological response of DIPG/GBM cell models to IL13Rα2 antibody-based agents. As a first-pass proof-of-concept experiment, we used a primary antibody + secondary antibody::cytotoxic agent system to test if IL13Rα2 expression conferred sensitivity to IL13Rα2 targeting agents. We selected four DIPG cell models and one GBM cell model (Table 1) for the proof-of-concept experiments. Cells were exposed to fixed concentrations of IL13Rα2 2E10 primary antibody in combination with varying concentrations of a secondary antibody conjugated to duocarmycin, a potent cytotoxic agent commonly conjugated to ADCs (Additional file 1: Fig. 1a). While the results did not show a direct relationship between IL13Rα2 expression and duocarmycin sensitivity, two models showed stronger response (SF-8628 and CHLA-200), demonstrating that IL13Rα2 expression can confer sensitivity to antibody-based therapies.

We theorized that the inability of IL13Ra2 antibody-mediated cytotoxin delivery to decrease cell viability in some models was due to failure of duocarmycin to overcome innate chemoresistance of most DIPG cell models. To interrogate this possibility, we selected a IL13Ra2-high and one IL13Ra2-low cell model (SF-8628 and DIPG-24, respectively) and treated them with two commonly used and readily accessible ADC payloads, DM-1 (also called mertansine) and monomethyl auristatin F (MMAF). SF-8628 was sensitive to both MMAF (IC_50_ = 3.84 µM, Additional file 1: Fig. 1b) and DM-1 (IC_50_ = 82.5 nM, Additional file 1: Fig. 1c), while DIPG-24 showed negligible sensitivity to (MMAF, Additional file 1: Fig. 1b) and slight sensitivity to DM-1 (Additional file 1: Fig. 1c). Disparity in cellular response to potent single agent cytotoxics suggests that selection of the payload agent may be critical for the effectiveness of any ADC developed to eliminate DIPG cells.

Partially positive results from the proof-of-concept experiments supported the continued evaluation of IL13Rα2 as a target for ADC therapies in DIPG; thus, we developed a fully conjugated antibody::cytotoxic agent for experimental validation. As previously mentioned, a critical challenge of DIPG treatment is the clinically-demonstrated resistance to most standard chemotherapeutic agents and the failure of most clinical trials [9,14,18,20,21,27,29,38], also reflected in the varied efficacy of the ADC cytotoxic agents tested on the DIPG cell models (Additional file 1: Fig. 1b, c). Thus, we selected pyrrolobenzodiazepine (PBD) as the payload for the IL13Rα2 ADC as PBD is known to promote cell death in model systems often resistant to standard chemotherapy agents or standard ADC cytotoxic payloads[39].

**Fig. 3:**
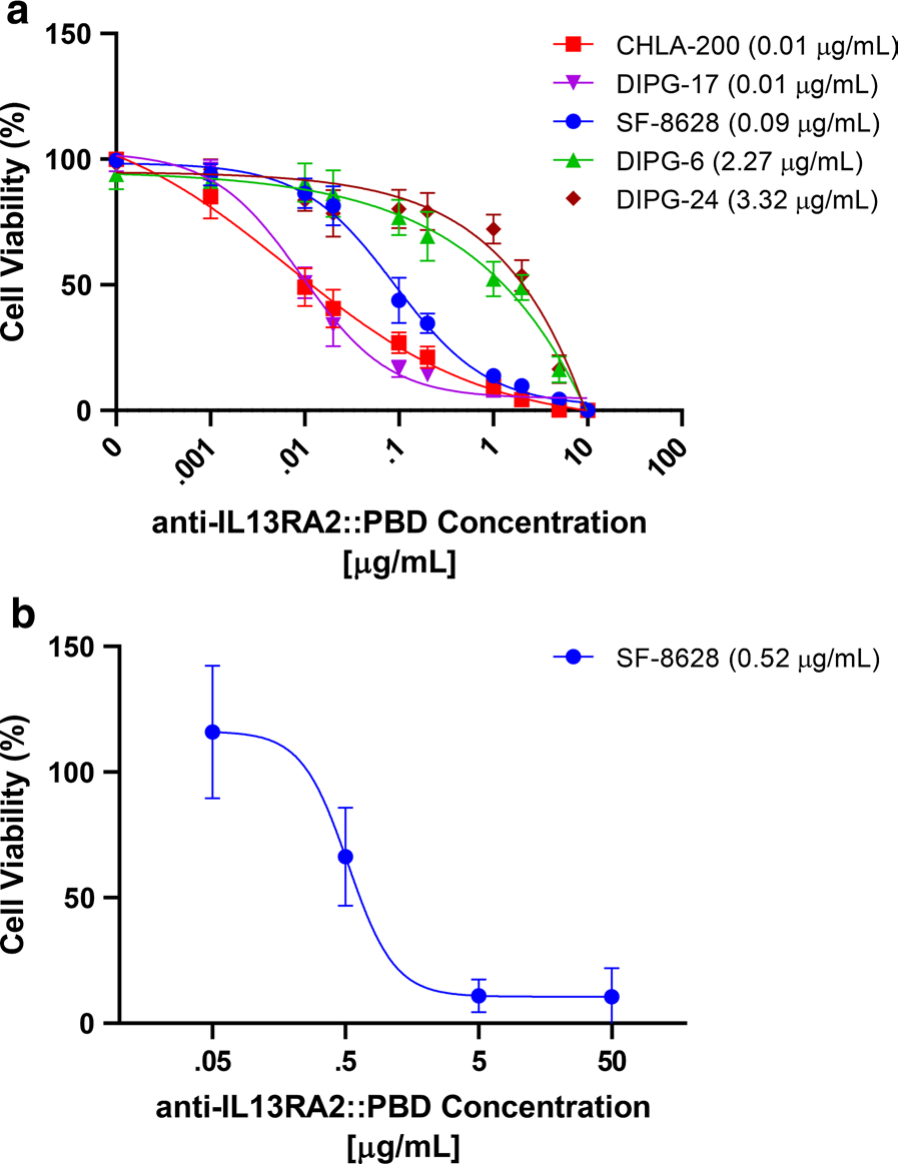
Results from anti-IL13Rα2::PBD ADC cell viability assays in DIPG/GBM cell models. **a** Cell viability following incubation with anti-IL13Rα2::PBD ADC for four DIPG cell models (DIPG-17, SF-8628, DIPG-6, DIPG-24) and one GBM cell model (CHLA-200). Values in parentheses are IC_50_ values. **b** Cell viability of SF-8628 ex ovo xenograft following anti-IL13Rα2::PBD ADC treatment. The value in parentheses is the IC_50_ value. Dose response points represent minimum n = 7 quail per concentration

We validated the activity of the anti-IL13Rα2::PBD ADC using the same cell models used in previous experiments (Table 1). Results from the cell viability assays demonstrate that three of four IL13Rα2-positive cell models (CHLA-200, DIPG-17, and SF-8628) were highly sensitive to anti-IL13Rα2::PBD, with all three cell models reaching 50% cell viability below 100 ng/mL ADC concentration and under 10% cell viability at highest tested ADC concentrations (Fig. 3a). One IL13Rα2-high cell model showed lesser response to the ADC (DIPG-6, IC_50_ = 2.27 µg/mL), which could potentially be attributed to DIPG-6 often demonstrating resistance to many agents[25]. The IL13Rα2-low cell model also demonstrated lesser response to the ADC (DIPG-24, IC_50_ = 3.32 µg/mL) consistent with the lower (but not absent) expression of IL13Rα2.

Finally, we tested the efficacy of the anti-IL13Rα2::PBD ADC in a luciferase/RFP-expressing SF-8628 quail *ex ovo* xenograft, with the ADC again showing strong response and a clear dose-dependent response (IC_50_ = 0.59 µg/mL) in the immunoprivileged *ex ovo* model system (Fig. 3b). Overall, anti-IL13Rα2::PBD drug response associates well (4/5 models tested) with IL13Rα2 expression in DIPG and positive response successfully translates to an ex ovo xenograft system.

## Discussion

Relatively few treatments exist for DIPG, with the majority of clinical trials having demonstrated little to no improvement in clinical outcomes. Previous investigations of IL13Rα2 as a therapeutic target by various treatment modalities [10,11,33,34] suggest the utility of IL13Rα2 targeting in the context of an immunotherapy or immunoconjugate for multiple diseases, including DIPG. However, overall clinical experience using CAR-T cell therapy in brain tumors has identified barriers to advancement. Specifically, the milieu of immune inflammatory responses to CAR-T cell therapy (termed CAR T‐related encephalopathy syndrome, CRES) are often life-threatening toxicities [1]. While ADC therapies can also potentially illicit immunogenic responses, clinical use of bevacizumab monoclonal antibody therapy in glioblastoma has demonstrated a reduced need for corticosteroids as a consequence of treatment [24] suggesting monoclonal antibodies (and by extension ADC agents) may have lessened immunogenicity in brain tumors compared to CAR-T cell therapy. Additionally, brain tumor microenvironments are frequently immunosuppressive [1], which may be less challenging to an ADC operating through cytotoxicity-induced apoptosis compared to CAR-T cell therapy which requires immune cell involvement.

From our studies, three key points can be taken from the exploration of IL13Rα2 as a therapeutic target in DIPG. First, stimulation by both IL13Rα2 cytokine binding partners (IL-4 and especially the canonical ligand IL-13) demonstrate negligible effect on cell proliferation or invasion, suggesting that IL13Rα2 binding and signaling may not be essential to DIPG progression, thus even inadvertent stimulation of this pathway through the use of an ADC is unlikely to negatively impact the course of treatment. Second, high expression of IL13Rα2 in DIPG and GBM associates strongly with sensitivity to anti-IL13Rα2::PBD ADC, although PBD may have limitations in killing more resistant DIPG cell models as PBD acts through inducing DNA damage [55]. Finally, as anti-IL13Rα2::PBD demonstrates efficacy in most but not all DIPG models, the selection of the cytotoxic payload will be critical for the success of any ADC developed for DIPG.

The negligible impact on either cell proliferation or invasion is somewhat surprising, given previous studies demonstrating IL13RA2 acting as a strong mediator of both proliferation and invasion in multiple diseases [4,23,43]. In glioblastoma, IL13RA2 has been shown to cooperate with mutant EGFRvIII to mediate growth [43], while EGFR mutations including EGFRvIII are believed to not be widely recurrent in DIPG[25, 53]which may limit the importance of IL13RA2 in DIPG progression. Additionally, a separate study performed in our laboratory explored the effect of multiple receptor-ligand pairs highly expressed in DIPG and known to impact proliferation in other disease, with the majority of canonical signaling pairs demonstrating similarly limited impact on DIPG cell proliferation and invasion (manuscript in preparation).

Despite the positive correlation between anti-IL13Rα2::PBD ADC sensitivity and IL13Rα2 expression status, DIPG-6 insensitivity to the ADC exemplifies the key challenge in designing ADC therapeutics for DIPG. A key requirement in the design of a clinical ADC is selecting an exceedingly potent cytotoxic capable of affecting cellular death at low (~ 10 nM) concentrations, as ADC efficacy is heavily linked to payload efficacy [42]. While the innate chemoresistance of DIPG may be somewhat abrogated by PBD’s ability to overcome chemoresistance, PBD still operates via DNA damage mechanisms similar to duocarmycin [55] and thus selecting a non-standard ADC payload may potentially overcome the resistance seen in DIPG-6 and potentially other DIPG cell models.

Nonetheless, the promising proof-of-concept results of anti-IL13Rα2::PBD ADC in DIPG present a translational opportunity. There are currently no clinically-validated therapeutics available for DIPG, nor are there any FDA approved IL13Rα2 immunoconjugate agents. Successful translation of an IL13Rα2 ADC for DIPG would provide a promising new therapeutic to a pediatric disease which lacks any viable life-saving treatment options and would enable broader clinical use of an immunoconjugate agent for a cell surface target overexpressed in numerous cancer types. Moving an IL13Rα2 ADC to clinical use will require optimizing the antibody binding dynamics and maximizing efficacy of the cytotoxic payload while minimizing peripheral toxicity, optimizations which are the requisite next steps for initiation of an anti-IL13Rα2 ADC clinical trial for DIPG. Fortunately, convection-enhanced delivery (CED) of an antibody is already known to be clinically feasible[3]. Overall, despite the potential limitations of PBD as the cytotoxic payload, anti-IL13Rα2 ADC agents show promise as a therapeutic strategy for an exceptionally intractable disease such as DIPG.

## Supplementary Information

**Additional file 1: Figure 1**. Cell model response to exploratory immunoconjugate experiments. Cell viability results following a primary antibody–secondary antibody immunoconjugate assay as well as two cytotoxic agents commonly used as ADC payloads. Cytotoxic agents were tested on SF-8628 and DIPG-24. Values in parentheses are IC_50_ values. **a)** Cell viability following two-step incubation of anti-IL13Rα2 primary antibody plus a duocarmycin-conjugated secondary antibody. **b)** Cell viability following treatment with unconjugated MMAF. **c)** Cell viability following treatment with unconjugated DM-1.

**Additional file 2: Figure 2**. Validation of mechanistic function of IL-4 and IL-13 recombinant proteins. Human alveolar rhabdomyosarcoma cell line RH30 was exposed to 20 ng/mL IL-4 or IL-13 recombinant protein for 60 min. STAT6 expression and phosphorylation was detected via western blot to confirm expected activity of recombinant proteins based on previously published studies [26]. Control RH30 demonstrates no phosphorylation of STAT6, while both IL-4 and IL-13 exposure induce STAT6 phosphorylation, as expected.

**Additional file 3: Table 1**. Table of information, properties, and clinical and genomic characteristics for cell lines used in this study.

## Data Availability

Transcriptome sequencing data is available through the Database of Genotypes and Phenotypes (dbGaP) under accession IDs phs000900.v1.p1 and phs001526.v1.p1, and through the European Genome-Phenome Archive (EGA) under accession ID EGAD00001006450.
