# Correction to: Design considerations of an IL13Rα2 antibody–drug conjugate for difuse intrinsic pontine glioma

**DOI:** 10.1186/s40478-021-01210-w

**Published:** 2021-06-25

**Authors:** Xiaolei Lian, Dina Kats, Samuel Rasmussen, Leah R. Martin, Anju Karki, Charles Keller, Noah E. Berlow

**Affiliations:** Children’s Cancer Therapy Development Institute, 12655 SW Beaverdam Road-West, Beaverton, OR 97005 USA

## Correction to: *acta neuropathol commun* 9, 88 (2021) https://doi.org/10.1186/s40478-021-01184-9

In the original publication [1] there was an incorrect funding acknowledgement. In this correction article the correct and incorrect funding acknowledgement are published. The original article has been updated.

**Incorrect funding**

This study was funded by a grant from the Matthew Larson Foundation for Pediatric Brain Tumors, as well as donations in honor of Calee, Caleb, Nicole, and Andrew.

**Correct funding**

This study was funded by a grant from the Matthew Larson Foundation for Pediatric Brain Tumors, as well as donations in honor of **Caleb**, Caleb, Nicole, and Andrew.
