# Supplementary figures and images for: Design considerations of an IL13Rα2 antibody–drug conjugate for diffuse intrinsic pontine glioma
Source: Acta Neuropathol Commun. 2021 May 17;9:88. doi: 10.1186/s40478-021-01184-9 (PMC8127302; doi:10.1186/s40478-021-01184-9)

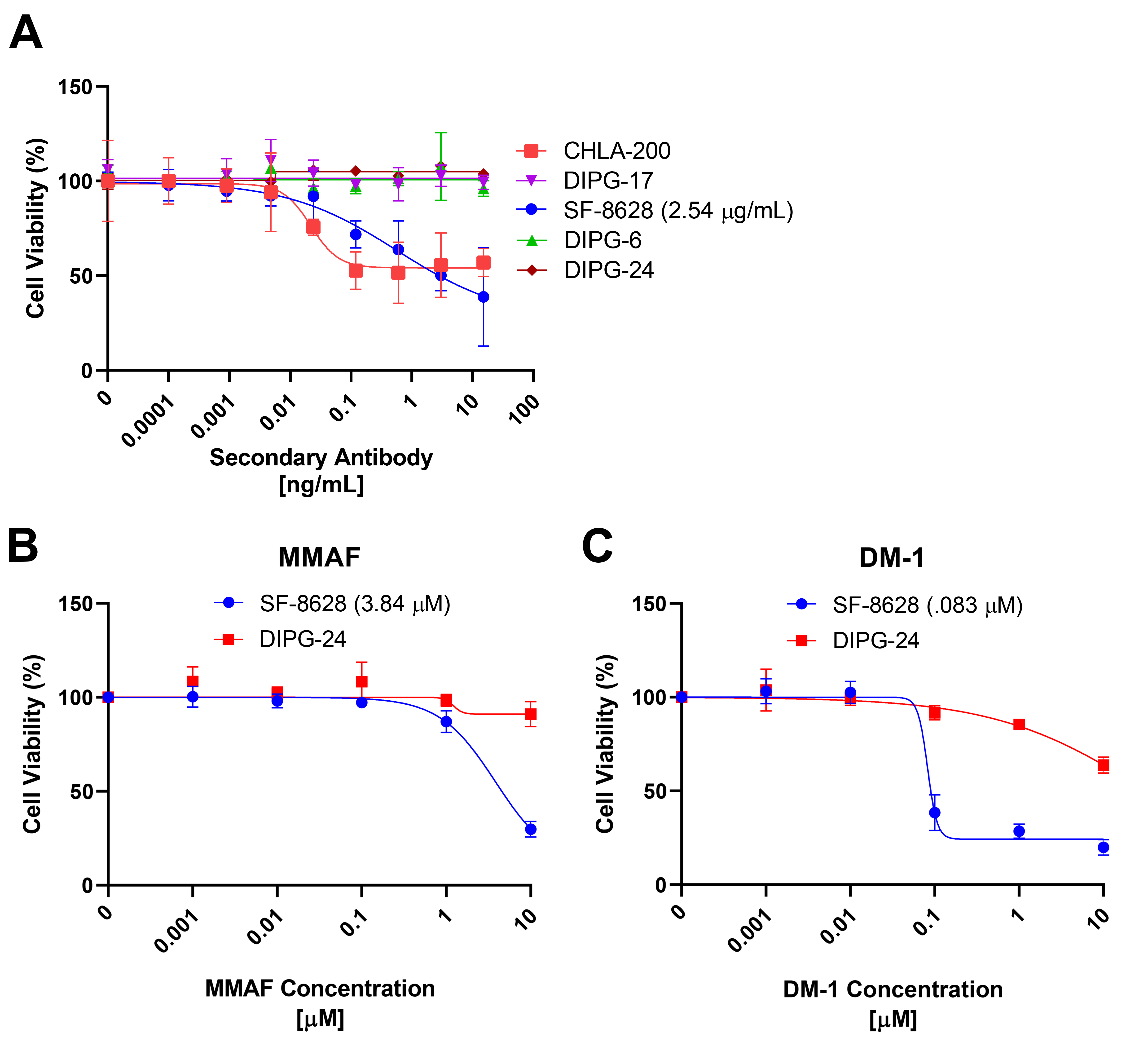

Supplement: Supplementary file 1 — Additional file 1: Figure 1. Cell model response to exploratory immunoconjugate experiments. Cell viability results following a primary antibody–secondary antibody immunoconjugate assay as well as two cytotoxic agents commonly used as ADC payloads. Cytotoxic agents were tested on SF-8628 and DIPG-24. Values in parentheses are IC50 values. a) Cell viability following two-step incubation of anti-IL13Rα2 primary antibody plus a duocarmycin-conjugated secondary antibody. b) Cell viability following treatment with unconjugated MMAF. c) Cell viability following treatment with unconjugated DM-1. [file 40478_2021_1184_MOESM1_ESM.tif]

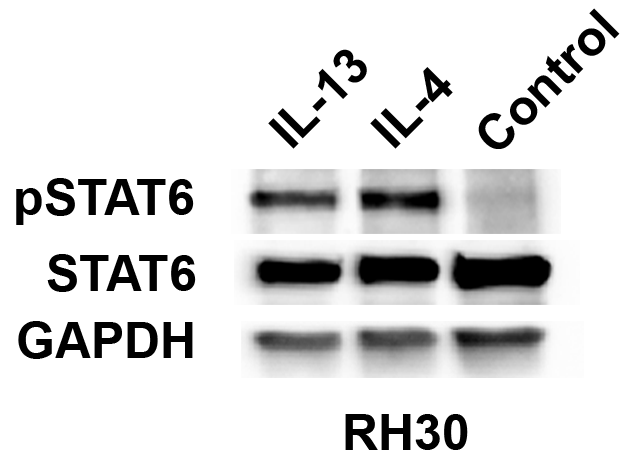

Supplement: Supplementary file 2 — Additional file 2: Figure 2. Validation of mechanistic function of IL-4 and IL-13 recombinant proteins. Human alveolar rhabdomyosarcoma cell line RH30 was exposed to 20 ng/mL IL-4 or IL-13 recombinant protein for 60 min. STAT6 expression and phosphorylation was detected via western blot to confirm expected activity of recombinant proteins based on previously published studies [26]. Control RH30 demonstrates no phosphorylation of STAT6, while both IL-4 and IL-13 exposure induce STAT6 phosphorylation, as expected. [file 40478_2021_1184_MOESM2_ESM.tif]
